# Complete Genomic Characterization of Porcine Reproductive and Respiratory Syndrome Virus Strain HB-XL

**DOI:** 10.3390/genes6030672

**Published:** 2015-07-23

**Authors:** Yi Zuo, Wanzhe Yuan, Jiguo Sun

**Affiliations:** 1 College of Animal Medicine, Agriculture University of Hebei, Baoding 071001, China; E-Mail: 15200087636@163.com or sjg2000@mail.hebau.edu.cn; 2 Hebei Engineering and Technology Research Center of Veterinary Biotechnology, Baoding 071001, China

**Keywords:** porcine reproductive and respiratory syndrome virus, Nsp2, GP5, variation analysis

## Abstract

Porcine reproductive and respiratory syndrome virus (PRRSV) is the causal agent of a serious disease of swine. Here, we report the genome sequence of PRRSV strain HB-XL isolated from a pig farm with a clinical outbreak of porcine reproductive and respiratory syndrome. The genome is 15,323 bp long and has nine open reading frames (GenBank: KP162169). Comparative and phylogenetic analysis showed that HB-XL belongs to the highly pathogenic PRRSV (HP-PRRSV) subfamily in the family PRRSV. The viral nonstructural protein 2 (Nsp2) of the HB-XL strain contained 30 discontinuous amino acid (AA) deletions relative to that of the Nsp2 of the VR2332 strain. The AA substitutions R13 and R151 suggested high virulence of the HB-XL strain. The unique mutations in glycoprotein 5 (GP5) and Nsp2 revealed that HB-XL might be a novel variant PRRSV strain recombined with vaccine strains. However, the low morbidity and mortality in the pig herd from which HB-XL was isolated indicate that the virulence of the virus was weak, so it has potential as a future vaccine strain.

## 1. Introduction

Porcine reproductive and respiratory syndrome virus (PRRSV) is the causative pathogen of porcine reproductive and respiratory syndrome (PRRS). Porcine reproductive and respiratory syndrome (PRRS) is currently a world-wide economically important disease in the global swine industry. PRRS has two major clinical manifestations: reproductive failure in sows including late-term abortions, increased chances of stillborns, mummified and weak-born pigs, and respiratory disease such as interstitial pneumonia in all ages of pigs [1]. Though it was first reported in the United States in 1987 and later in Europe [2,3], gradually, hog-rearing Asian nations were impacted as well. In 2006, an emerging highly pathogenic strain of porcine reproductive and respiratory syndrome virus (PRRSV), which causes continuous high fever and a high proportion of deaths in vaccinated pigs of all ages, broke out in mainland China and spread rapidly to neighboring countries, causing enormous economic losses [4,5,6]. PRRSVs are geared for rapid variation through mutation or recombination [7,8], resulting in new isolates with different levels of pathogenicity and virulence. Therefore, it is of great scientific significance to explore the molecular pathogenesis of highly pathogenic PRRSV (HP-PRRSV) emerging in China.

PRRSV is an enveloped, single-strand, positive RNA virus which belongs to the family Arteriviridae of the order Nidovirales, which includes members of the Coronaviridae and Roniviridae families [2]. The genome of PRRSV is approximately 15 kb in size and is capped at the 5'-end and polyadenylated at the 3'-terminus [9]. It contains at least nine overlapping open reading frames (ORFs) [10]. ORF1a and ORF1b are located downstream of the 5'-untranslated region (UTR) and encode the viral nonstructural proteins (Nsps): Nsp1α, Nsp1β, and Nsp2 to −12 [11]. ORF2 to −7 are located at the 3'-end of the genome and encode the viral structural proteins GP2, E, GP3, GP4, GP5, M, and N [12]. PRRSV was classified into two major genotypes, represented by the North American prototype VR-2332 and the European prototype Lelystad virus (LV). The two genotypes exhibit distinct genetic variations, with approximately 60% nucleotide identity at the genome level [13]. Furthermore, strains within each genotype vary considerably, the coding region for the nonstructural protein 2 (Nsp2) and ORF5 of PRRSV display substantial genetic variation [14]. Thus, ORF5 and Nsp2 have become the regions of choice for monitoring the evolution of PRRSV and for molecular epidemiology research on PRRSV [15].

To these ends, the complete genome of PRRSV strain HB-XL isolated from one pig farm with clinical outbreak of porcine reproductive and respiratory syndrome (PRRS) was sequenced and analyzed. The study revealed the genotype and the genomic characteristics of the HB-XL strain, and enriched genomic data of PRRSV.

## 2. Materials and Methods

**Figure 1:**
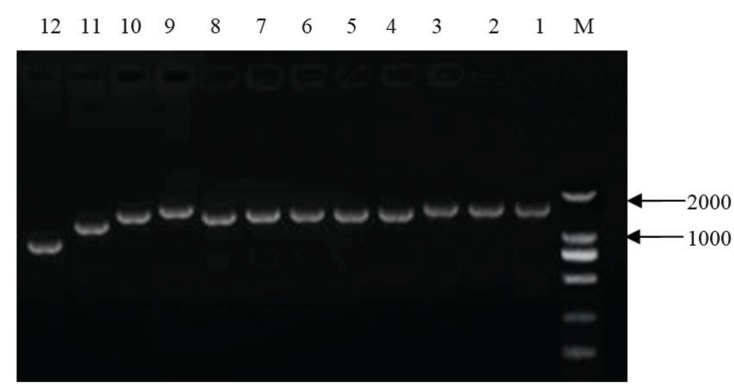
PCR amplification on the complete gene of HB-XL strain M, DL2000Maker; 1–12, complete gene of the HB-XL strain.

PRRSV strain HB-XL, used in this study, was originally isolated from swine cultivated in Hebei, China. Total nucleic acid was extracted from the cell culture supernatant of a single passage using the Viral RNA Kit (Tiangen, Beijing, China), used for reverse transcription and PCR amplification with PrimeSTAR^HS^ polymerase(Takala). Twelve pairs of specific primers were designed in Table 1 to obtain the full-length sequence of HB-XL. The PCR was done under the following conditions in a thermal cycler: 1 cycle of 3 min at 98 °C; 33 cycles of denaturation at 94 °C for 30 s, annealing at 55 °C for 30 s, and elongation at 72 °C for 1 min 37 s; and 1 cycle of 10 min at 72 °C. The amplified products were analyzed by electrophoresis in a 1% agarose gel. The 12 fragments of the complete genome of HB-XL were termed seq1–seq12 (0.8 kb–1.5 kb), respectively, according to their sizes (Figure 1). PCR products were excised and the gel purified using the PureLink Quick Gel Extraction Kit (Tiangen, Beijing, China). The products were subcloned into pEASY-Blunt cloning vectors (Trangen, Beijing, China) and subsequently subjected to Sanger Sequencing reactions (Invitrogen, Beijing, China). Genomic analyses were conducted using the ClustalW program in the DNAStar. Phylogenetic trees were constructed using the distance-based neighbor-joining method in the MEGA 5.

**Table 1:** Primers used for PCR for HB-XL.

Primers Name	Sequence (5'–3')	Position ^a^
PRRSV1F	ATGACGTATAGGTGTTGGCTCTATG	1–1562
PRRSV1R	AGGGTGGTCTCAAAGTTGGAAT	
PRRSV2F	ATTTCCGCCATCGTCAACCG	1513–3040
PRRSV2R	TCTGGTGCGTCAGCGTTGTTGT	
PRRSV3F	ACACCTATGAGTGAGCCCGTAC	2827–4324
PRRSV3R	CAACAATACCAAGCCTAAGCAA	
PRRSV4F	TGCTTAGGCTTGGCATTGTTG	4304–5656
PRRSV4R	TGACGGTGTTCAGTGAGGGCT	
PRRSV5F	CACCGCACCAGATGGAACCTACT	5427–6890
PRRSV5R	GTGCCTCGGACCTTATCAACCTGT	
PRRSV6F	AGTGCTTTGTTTCTGCGTCCAAC	6770–8241
PRRSV6R	GAATGGTCGGCACATACAACTCA	
PRRSV7F	CCTGCCTCACGCCTAATGCTA	8233–9638
PRRSV7R	CCCGCACATTCTGGACTTCTTC	
PRRSV8F	TTCCTGGCCCACCGTTCTTCTT	9559–10861
PRRSV8R	AGAGCCTGAGCAACCGTGATTT	
PRRSV9F	GCAAGACATGCTATCTTCGTGT	10704–12151
PRRSV9R	ATGGACACCAGAAATTCCGTGA	
PRRSV10F	CCCTGTCATTGAACCAACTTTA	12035–13533
PRRSV10R	AGCATGAGGAGGTCAGAAGAAT	
PRRSV11F	AACACCACCGCAGCATCAAACT	13346–14685
PRRSV11R	CCTAGCAAGCACAAACGGCATC	
PRRSV12F	TTCAGAGCACAAATAGGGTCGCG	14565–15408
PRRSV12R	TAATTGCGGCCGCATGGTTCTC	

^a^ Numbers represents the nucleotide position within the genome of BD2 (GenBank acession number: KF709977.1).

## 3. Results

### 3.1. Genomic Characteristics on the HB-XL Strain

The sequence analysis showed that the full-length genomic sequence of HB-XL was 15,323 nucleotides in length, excluding the polyadenylated sequences, and included the following UTRs: 5'-UTR(1–188), 3'-UTR(15171–15320). It may contains these overlapping open reading frames (ORFs): ORF1a(189–7610), ORF1b(7608–11981), ORF2(11983–12753), ORF3(12606–13370), ORF4(13151–13687), ORF5(13698–14300), ORF6(14285–14809), ORF7(14799–15170); the estimated molecular weight of GP2, E, GP3, GP4, GP5, M, and N were 28.2kD, 10kD, 28.0kD, 19.7kD, 22.1kD, 19.2kD, 13.6kD, respectively.

### 3.2. Homology Analyses

The nucleotide sequence and amino acid sequence of HB-XL was compared with those of the other PRRSV isolates, including one North American strain (VR-2332), one European strain (LV), and some Chinese strains (Table 2). Sequence data were analyzed by using DNAStar. Multiple-sequence alignments were done by using ClustalW.

**Table 2:** Representative PRRSV strains used in this study.

No.	Name	Country	Year	Accession Number
1	CH-1a	China	1996	AY032626
2	BJ-4	China	2000	AF331831
3	HB-1(sh)/2002	China	2002	AY150312
4	JXA1	China	2006	EF112445
5	HUB2	China	2006	EF112446
6	HuN4	China	2007	EF635006
7	SY0608	China	2007	EU144079
8	CH-1R	China	2008	EU807840
9	HB-1	China	2008	EU360130
10	WUH4	China	2011	JQ326271
11	QYYZ	China	2011	JQ308798
12	SD16	China	2012	JX087437
13	JXA1-R	China	2012	JQ804986
14	HEB-2013	China	2013	KJ591659
15	HB-XL	China	2013	KP162169
16	LMY	South Korea	2002	DQ473474
17	VR-2332	USA	1992	AY150564
18	EuroPRRSV	USA	1999	AY366525
19	RespPRRS MLV	USA	1994	AF066183
20	Lelystad virus	Europe	1991	M96262

The HB-XL strain shared a higher level of nucleotide sequence identity with the Type-2 PRRSV strains (87.7% to 99.1%) compared to that shared with the Type-1 PRRSV strain LV (61.7%). The 5'-UTR and 3'-UTR of HB-XL shared nucleotide sequence identities of 90.7% to 97.4% with the Type-2 PRRSV strains, compared to that shared with the Type-1 PRRSV strain LV (50.1% and 55.9%) and the recombination strain QYYZ (95.2% and 87.3%). The ORF1a and ORF1b of HB-XL shared nucleotide sequence identities of 87.5% and 99.4% with those of Type-2 PRRSV strains, respectively, whereas they shared 59.3% and 61.5% with those of the LV strain, respectively. The Nsp2 of HB-XL exhibited the highest levels of variability, the predicted AA sequences of the Nsp2 of HB-XL shared 97.1% identity with the JXA1 strain, with 30.0% and 74.2% nucleotide sequence identities shared with the LV and QYYZ strains, respectively. The comparisons of the sequences of ORF2–7 showed that the HB-XL strain shared 88.9% to 99.8% nucleotide sequence identity with the Type-2 PRRSV strains, compared to that shared with the Type-1 PRRSV strain LV (63.7% to 69.2%) and the recombination strain QYYZ (83.9% to 91.0%). The amino acid sequence identity shared between the HB-XL and Type-2 PRRSV strains ranged from 86.6% to 100%. These results indicated that the HB-XL strain is highly similar to a group of highly pathogenic (HP) strains of PRRSV previously isolated in China. The highest level of shared nucleotide sequence identity (99.1%) was observed between the HB-XL strain and the JXA1, which had previously caused epidemics in China (Table 3).

**Table 3:** Nucleotide and deduced amino acid identities of HB-XL compared with those of HB-1(sh)/2002, JXA1, CH-1a, QYYZ, VR-2332, and LV (%).

HB-XL %	Identity to SH1211					
	HB-1(sh)/2002	JXA1	CH-1a	QYYZ	VR-2332	LV
Nucleotides (length)						
°5'UTR (188)	96.8	97.4	97.3	95.2	93.1	50.1
°ORF1a (7422)	96.6	99.1	94.0	85.2	87.5	59.3
°ORF1b (4383)	97.5	99.4	92.8	90.3	91.1	61.5
°ORF2 (771)	96.2	98.6	94.7	90.0	93.1	67.1
°ORF3 (759)	95.4	98.8	92.9	90.8	88.9	61.4
°ORF4 (537)	96.8	98.7	93.9	94.4	89.9	67.6
°ORF5 (603)	96.5	99.0	95.0	83.9	88.9	63.7
°ORF6 (525)	97.1	99.8	96.3	91.0	95.6	69.2
°ORF7 (372)	96.5	99.7	94.8	89.5	94.4	65.9
°3'UTR (150)	93.3	97.3	93.7	87.3	90.7	55.9
Complete (15,323)	96.9	99.1	95.0	87.7	89.5	61.7
Amino acid (length)						
°Nsp2 (950)	91.1	97.1	84.9	74.2	74.8	30.0
°GP2 (257)	94.5	97.7	94.9	89.5	92.2	63.1
°E (74)	94.5	97.5	94.5	89.0	92.0	63.0
°GP3 (253)	92.9	93.8	89.3	87.4	88.6	54.9
°GP4 (179)	97.8	96.1	97.8	94.9	90.4	70.9
°GP5 (201)	92.5	97.5	91.0	81.6	86.6	56.2
°M (175)	98.3	99.4	98.3	96.6	98.3	79.8
°N (124)	95.9	100	95.1	91.9	95.1	58.1

### 3.3. Phylogenetic Analyses

**Figure 2:**
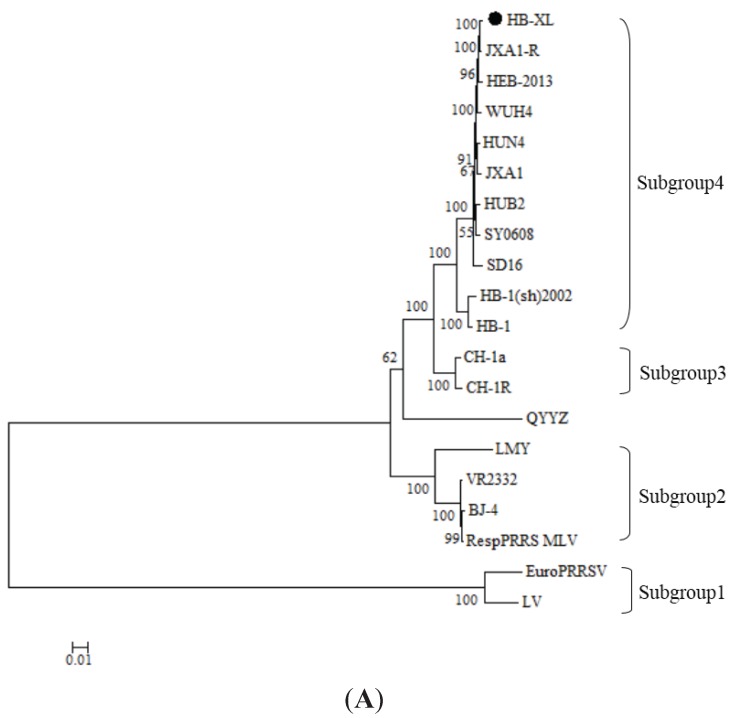

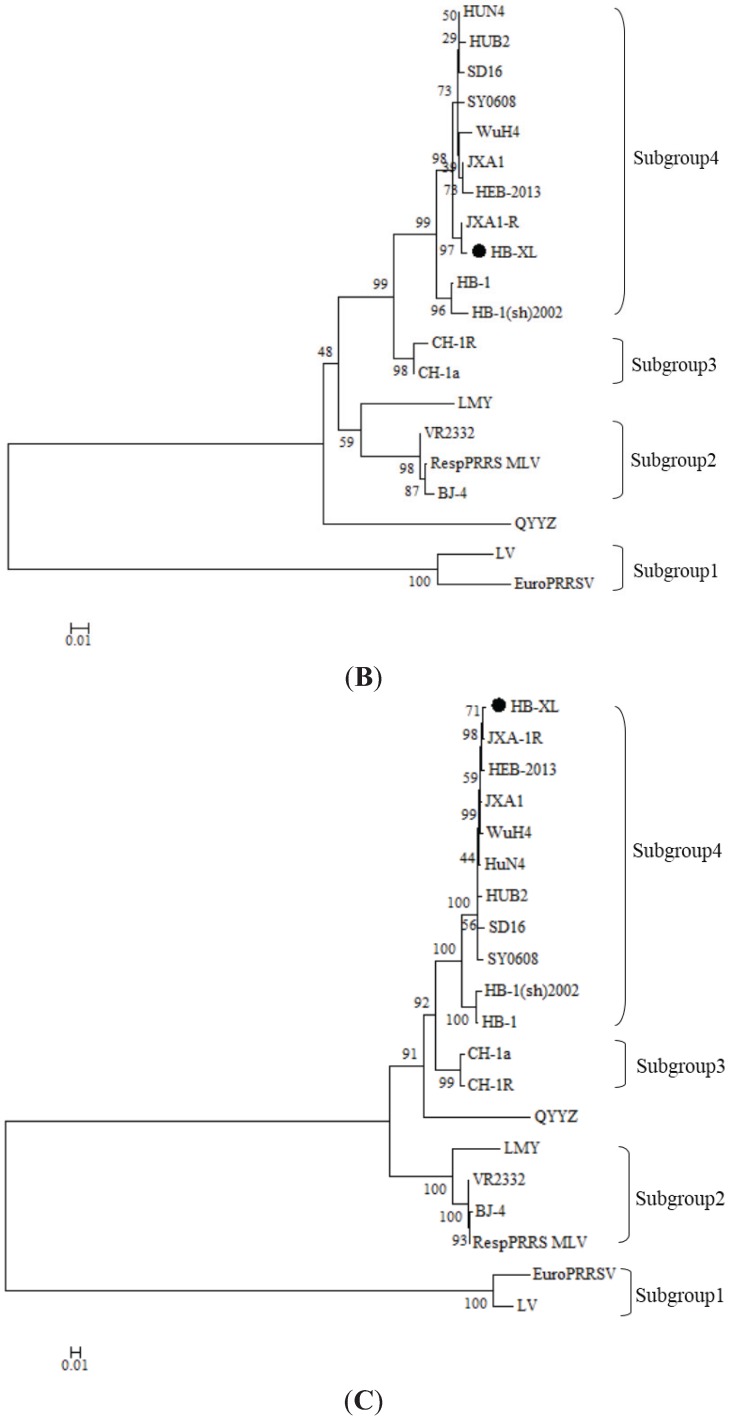
Phylogenetic trees based on the complete genome, ORF5, and Nsp2 of PRRSV. (**A**) Complete genome; (**B**) Open reading frame (ORF); (**C**) Nsp2 nucleotide. The multiple sequence alignment was obtained using the MEGA5 program, and the phylogenetic tree was constructed by the neighbor-joining method using 1000 bootstrap replicates of the sequence data. Bootstrap confidence limits are shown at each node. The isolate identified in this study is indicated by black dots.

The phylogenetic relationships were studied by using MEGA5 software according to the nucleotide sequences of HB-XL and other known PRRSV isolates worldwide (Figure 2). Phylogenetic trees were generated on the nucleotide sequences of the complete genome, Nsp2, and ORF5 of HB-XL, and the various PRRSVs. The topology of trees based on the complete genome, Nsp2, and ORF5 nucleotide sequences revealed that all PRRSV isolates belonged to four main groups (Groups 1–4). Group 1 comprises European strains (LV and EuroPRRSV). Group 2 contains four North American strains (VR2332, RespPRRS MLV, BJ-4, and LMY). One recombination strain (QYYZ) was independent of one branch. Group 3 contains several classical Chinese strains (e.g., CH-1a and Ch-1R). Group 4 contains some Chinese HP-PRRSV strains (e.g., JXA1 and WUH4). The phylogenetic tree based on complete genome, ORF5, and Nsp2 nucleotide sequences revealed similar relationships among the various PRRSV strains, with HB-XL forming Subgroup 4. It also showed that the HB-XL strain is closely related to the Chinese isolate JXA1-R strain.

### 3.4. Variation Analyses

**Figure 3:**
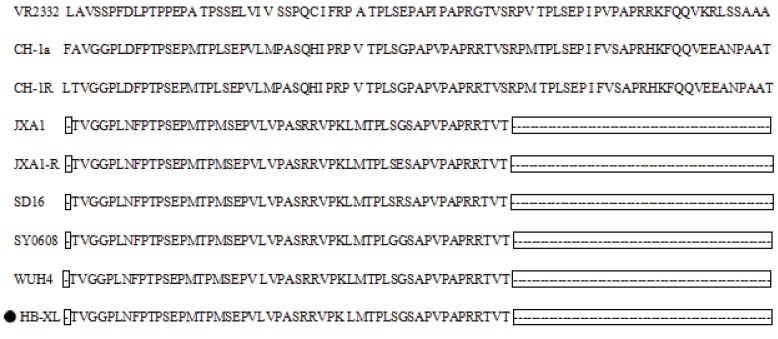
Alignment of the partial Nsp2 amino acid sequence of HB-XL and several representative PRRSV strains with VR2332. The amino acid deletions are shown with lines. The isolate identified in this study is indicated by a black dot.

The coding region for the nonstructural protein 2 (Nsp2) and ORF5 of PRRSV displays substantial genetic variation. The 950-AA sequence of the protein produced from the Nsp2 gene of the HB-XL strain contained 1 and 29 noncontiguous AA deletions at positions 481 and 533 to 561, relative to that of the Nsp2 gene of the VR2332 strain. These results indicated that the HB-XL strain is highly similar to a group of highly pathogenic (HP) strains of PRRSV previously isolated in China (Figure 3). In addition, the AA mutations in position 11, 161, 51, and 994 were found in Nsp2 of the HB-XL, compared other PRRSV strains. Residues 13 and 151 in the GP5 proteins of PRRSVs have been shown to be associated with virulence [16]. The AA substitutions R^13^ and R^151^, which were identified in the predicted GP5 protein of the HB-XL strain, suggested high virulence of the HB-XL strain. The mutations at G^33^ (N^33^ → G^33^) and N^54^ (D^54^ → N^54^) were found in the predicted GP5 protein of HB-XL, relative to that of other PRRSVs examined. To gain further insight into the genetic evolution of HB-XL, we also analyzed variation in potential *N-*glycosylation sites by NetOGlyc 4.0 Server. There were four conservative *N*-glycosylation sites (30, 33, 44, 51) in GP5 [17]. The GP5 of the HB-XL strain shared five *N*-glycosylation sites at AA positions 30, 34, 35, 44, and 51. The addition of two *N-*glycosylation sites at positions 34 and 35, and the deletion of one *N-*glycosylation site at position 33 was identified, relative to those in the GP5 protein of the VR2332 and JXA1 strains (Table 4). In addition, the predicted GP5 protein of the HB-XL strain has a similar mutation in site 33 with the QYYZ strain.

**Table 4:** *N*-glycosylation sites in PRRSV strains.

Strain Name	*N*-Glycosylation Sites						
	30	32	33	34	35	44	51
CH-1a	N	N	S	N	S	N	N
BJ-4	N	S	N	D	S	N	N
HB-1(sh)/2002	N	S	N	N	S	N	N
JXA1	N	S	N	N	N	N	N
HUB2	N	S	N	N	N	N	N
HuN4	N	S	N	N	N	N	N
SY0608	N	S	N	N	N	N	N
CH-1R	N	N	S	N	S	N	N
HB-1	N	S	N	N	N	N	N
WUH4	N	S	N	N	N	N	N
QYYZ	S	N	G	N	S	N	N
SD16	N	S	N	N	N	N	N
JXA1-R	N	S	D	N	N	N	N
HEB-2013	N	S	N	N	N	N	N
HB-XL	N	S	G	N	N	N	N
LMY	S	N	N	S	S	N	N
VR-2332	N	S	N	D	S	N	N
EuroPRRSV	N	S	N	D	S	N	N

## 4. Discussion

The PRRS continues to be a serious threat, causing a significant economic impact on the swine industry worldwide [18]. Although commercial vaccines against PRRSV are available, traditional control strategies and conventional vaccines have failed to provide sustainable disease control. Surveillance of the recently emerged strains has become necessary because of the considerable genetic and antigenic diversity identified in these HP-PRRSV isolates [15]. In our current study, a novel variant PRRSV strain was isolated from a piglet in a PRRSV-vaccinated pig herd with high morbidity and mortality in Hebei, China.

The HB-XL was 15,323 nucleotides in length. Comparative analysis of the nucleotides revealed that the virus shared 87.7%–99.1% identity with the representative strains of PRRSV, but only 61.7% with the type 1 virus LV, indicating that this new Chinese isolate was closely related to the type 2 North American PRRSV genotype. However, several unique mutations were found in HB-XL.

PRRSV undergoes remarkable genetic alterations. The Nsp2 gene has the highest genetic diversity in the genome of PRRSV [13]. It is, therefore, one of the main targets used to monitor PRRSV evolution [19]. Furthermore, the Nsp2-coding region is crucial for viral replication and the modulation of host immunity because of its protease activity [20]. Natural insertions and/or deletions are routinely observed in the hyper-variable middle region of the Nsp2 protein in field strains, resulting in polymorphism of considerable size [21]. The HB-XL contains discontinuous deletions of 30 amino acids in Nsp2. Although the 30-amino-acid deletion is not associated with the virulence of the emerging HP-PRRSV [22], it has been used as an epidemiological genetic marker for the dominant PRRSV in China since 2006. However, it indicated that a novel variant strain of PRRSV was identified in Hebei, China. Other AA mutations were found in Nsp2 of the HB-XL. Because the virulence of PRRSV is considered to be associated with multiple factors, whether such mutations were related with the virulence depends on further study.

In addition to the study of mutations and deletions in Nsp2, the genetic diversity of GP5 has received much attention because GP5 is the most important immunogenic protein and contains the primary neutralization epitope of PRRSV [23]. Furthermore, GP5 is one of the most genetically variable structural proteins of PRRSV. Thus, GP5 has also been a target for analysis of the genetic diversity of PRRSV [24]. From our analysis of residues 13 and 151 of GP5, we concluded that HB-XL was most closely related to the currently dominant HP-PRRSV. The primary neutralizing epitope (PNE) is also an important domain of GP5 regarding virus neutralization, and the H38(L/F)39 residues in this domain are considered to be critical to the immunogenicity of this epitope [25]. The AA Mutation L39I was observed in the PNE of HB-XL, which probably contributed to the ability of escaping neutralizing antibodies induced by PRRSV vaccines used in China, including the attenuated vaccine strains CH-1R and JXA1-R, which were derived from JXA1. In addition, a similar mutation in position 33 was observed in HB-XL and QYYZ [16]. A unique mutation in position 54 was identified in HB-XL. Whether this mutation is associated with virulence remains to be studied further. The high levels of shared nucleotides and amino acids sequence identities were observed between the HB-XL and the JXA1-R strains. Thus, HB-XL might be a novel variant of PRRSV strain recombined with a vaccine strains.

The decoy epitope of GP5 is comprised of (A/V)27LVN near the PNE, and it may delay the production of neutralizing antibodies of the virus [25]. However, no AA mutations were found in the decoy epitope of the GP5 of the HB-XL strain. Differences at the potential *N*-glycosylation sites were also observed in the GP5s of HB-XL and other isolates. Additional *N*-glycosylation sites may lead to an increase in the number of the *N*-linked glycans, providing a barrier to antibody evasion, which, in this case, does not affect the ability of the virus to bind to cellular receptors in the host [26]. The evolving glycan shield, therefore, presents a possible mechanism of a persistent increasing antibody repertoire of the virus [23].

The distribution of PRRSV has its own features [27]. The European strains usually emerged in European countries such as the Netherlands, Denmark, and UK. The North American strains emerged in North American and Asia. Highly pathogenic strains of PRRSV and classical strains of PRRSV co-existed in local pig farms and the majority of the viruses belong to HP-PRRSV. HP-PRRSV continues to undergo its genomic divergence, although it has not broken out in large-scale in recent years. Rapid evolution of PRRSV has been seen in China. The high degree of genetic and antigenic diversity among field isolates underlines the complexity of the control and eradication of the disease in China. Therefore, it is important to explore the molecular pathogenesis of HP-PRRSV emerging in China. However, the low morbidity and mortality in the HB-XL infected herd indicate that the virulence of the virus was weaker. It could be a vaccine strain in the future. Further studies are warranted to determine whether the AA variations in the Nsp2 and GP5 affect the replication, transcription, and virulence of the HB-XL strain. An understanding of all of these factors will be helpful in understanding the evolutionary characteristics of Chinese PRRSV and in planning the development and use of vaccines against PRRSV in the future.

## 5. Conclusions

The HB-XL strain represents a recently emerging virus with a genome structure that is typical of PRRSVs with unique genetic variation, including the unique mutations in GP5 and Nsp2. Our sequence analysis of the HB-XL strain provides insight into the role of the genetic variation of PRRSVs in China. Future studies of the immunogenicity and pathogenicity of the HB-XL strain are warranted to identify the mechanisms underlying the contribution of genetic variation in PRRSVs to host-pathogen interactions.
